# Differential Effects of Orientation and Spatial-Frequency Spectra on Visual Unpleasantness

**DOI:** 10.3389/fpsyg.2020.01342

**Published:** 2020-06-16

**Authors:** Narumi Ogawa, Isamu Motoyoshi

**Affiliations:** Department of Life Sciences, The University of Tokyo, Tokyo, Japan

**Keywords:** orientation, spatial frequency, visual unpleasantness, image statistic, Fourier spectrum

## Abstract

Increasing psychophysical evidence suggests that specific image features – or statistics – can appear unpleasant or induce visual discomfort in humans. Such unpleasantness tends to be particularly profound if the image’s amplitude spectrum deviates from the regular 1/*f* spatial-frequency falloff expected in natural scenes. Here, we show that profound unpleasant impressions also result if the orientation spectrum of the image becomes flatter. Using bandpass noise with variable orientation and spatial-frequency bandwidths, we found that unpleasantness ratings decreased with spatial- frequency bandwidth but increased with orientation bandwidth. Similarly, a subsequent experiment revealed that sinusoidal modulations in the amplitude spectrum of 1/*f* noise along the spatial frequency increased unpleasantness, but modulations along the orientation decreased it. Given that natural scenes tend to have a linear slope along the spatial frequency but an uneven spectrum along the orientation dimension, our opposing results in the spatial-frequency and orientation domains commonly support the idea that images deviating from the spectral regularity of natural scenes can give rise to unpleasant impressions.

## Introduction

Humans tend to prefer things that appear pleasant or clean to those that appear unpleasant or dirty. While such affective responses are usually evoked by the recognition of object category (e.g., rotten food) learned through daily experiences (Coon and Mitterer, 2012), affect may also be summoned by the visual appearance of the image itself. For example, it has been suggested that particular combinations of colors appear more or less pleasant (Ostwald, 1969). Arguably, humans may have a general preference for specific patterns, colors, and combinations thereof.

A number of psychophysical studies have demonstrated that visual stimuli with particular Fourier spectra can evoke feelings of discomfort or even induce severe negative emotions (Fernandez and Wilkins, 2008; Juricevic et al., 2010; Cole and Wilkins, 2013; Penacchio and Wilkins, 2015). Typically, normal observers report discomfort with images whose Fourier amplitude spectrum has a peak at spatial frequencies around 1–3 c/deg and that therefore deviates from the so-called 1/*f*^α^ spectral falloff that is characteristic of natural scenes (Wilkins et al., 1984; Wilkins, 1995; Fernandez and Wilkins, 2008; O’Hare and Hibbard, 2011). Indeed, an image including such a middle-frequency spectral peak – a cluster of holes and granules, for example – appears very uncomfortable to many people and can even induce pathological fear known as trypophobia (Cole and Wilkins, 2013; Le et al., 2015). A recent analysis with hundreds of natural surface images also indicates that a concentration of power in the middle spatial-frequency range is highly correlated with the unpleasantness of various surface materials including human skin (Otaka et al., 2019). Conversely, art images with a particular fractal dimension (Pentland, 1984; Spehar et al., 2003; Hagerhall et al., 2004; Taylor et al., 2005; Viengkham and Spehar, 2018) or a 1/*f*^α^ spatial-frequency spectrum (Graham and Field, 2007; Redies et al., 2007) are preferred, and a small proportion of unpleasant paintings have a roughly 2-octave spectral peak near 3 c/deg (Fernandez and Wilkins, 2008).

Further studies have investigated these effects by parametrically manipulating the spatial frequency spectrum in visual stimuli consisting of random noise (Conlon et al., 2001; Juricevic et al., 2010; O’Hare and Hibbard, 2011). For example, a 1/*f* noise image was rated as “uncomfortable” if a bump was added in the 0.4–1.5 c/deg spatial-frequency range (O’Hare and Hibbard, 2011). The same has been reported for images in visual arts (Fernandez and Wilkins, 2008). Other lines of the study showed that the spectral slope (α) of the log-log plot of natural scenes tends to be linear (Burton and Moorhead, 1987; Field, 1987; Tolhurst et al., 1992; Van Der Schaaf and Van Hateren, 1996), and examined the effect of the spectral slope of 1/*f* noise on rating (Juricevic et al., 2010; Spehar and Taylor, 2013; Spehar et al., 2015, 2016; Viengkham and Spehar, 2018; Viengkham et al., 2019). These findings largely support the same hypothesis that preference for 1/*f* noise images peaks for intermediate spectral slopes (α∼ = 1) and falls for slopes that deviate from the 1/*f* statistics.

Orientation is one of the most representative image features analyzed by the early visual system (Hubel and Wiesel, 1959; Bradley et al., 1987), but how the orientation characteristics of the image affect visual discomfort/unpleasantness has largely been ignored. There is evidence that natural scenes rarely have flat orientation spectra; instead, natural scenes tend to concentrate energy along the horizontal and vertical orientations with respect to the upright angle (Switkes et al., 1978; Van Der Schaaf and Van Hateren, 1996; Hansen and Essock, 2004). For close-up image of surfaces, natural lighting on 3D objects tend to produce oriented (not necessarily vertical or horizontal) rather than isotropic shading patterns. Assuming that unnatural image characteristics induce visual discomfort / unpleasantness, a flat orientation spectrum may therefore give rise to an unpleasant visual impression.

To examine if stimuli with a flat orientation spectrum tends to be judged as unpleasant/uncomfortable, the present study investigated unpleasantness ratings for bandpass filtered noise whose orientation and spatial frequency spectrum was parametrically manipulated. By using bandlimited noise with variable orientation and spatial-frequency bandwidths (Exp. 1) and noises modulated sinusoidally along either the orientation or spatial-frequency (Exp. 2), we show that human observers give larger unpleasantness ratings to images with flat orientation spectra or bumpy spatial-frequency spectra.

## Experiment 1

### Methods

#### Observers

Eleven naïve paid volunteers and one of the authors (NO) took part in the experiment (6 females and 6 males, 11/12 were students, aged 18–33). None had a history of migraines. All observers had normal or corrected to normal vision. All the experiments followed the Declaration of Helsinki guidelines and were conducted with permission from the Ethics Committee of the University of Tokyo. All observers provided written informed consent.

#### Apparatus

Visual stimuli were generated by a PC (DELL Precision T1600) and displayed on a gamma-corrected LCD monitor (BenQ XL-2730Z) with a 60 Hz refresh rate. Pixel resolution was 1.07 min/pixel at a viewing distance of 75 cm. All experiments were conducted in a dark room.

#### Stimuli

**FIGURE 1:**
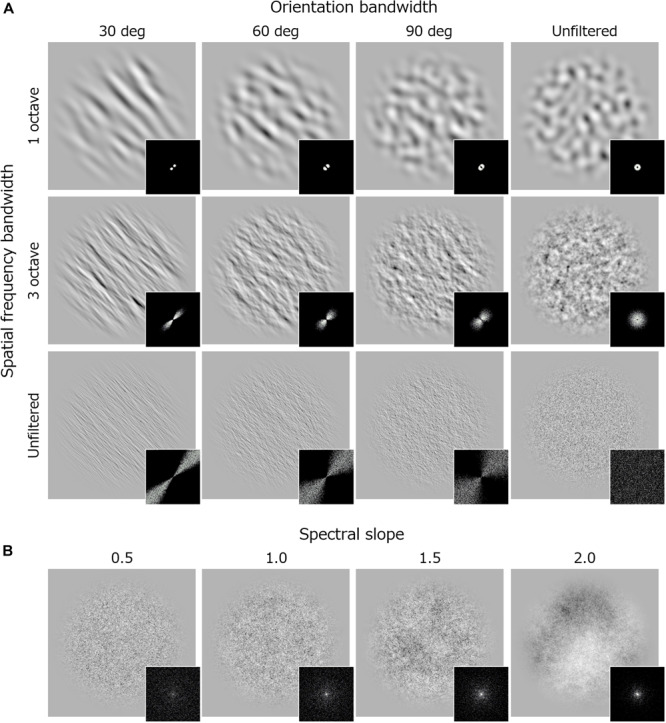
Examples of stimuli and their Fourier spectra used in Experiment 1. **(A)** Band-pass noise with spatial frequency bandwidth of 1 octave (upper), 3 (middle) octave, and unfiltered noise (lower). Each column shows stimuli with different orientation bandwidth. **(B)** 1/*f* noise with various spectral slope.

Visual stimuli were bandpass noise textures (Figure 1) – white noise filtered by a Gaussian filter with a particular spatial frequency and orientation bandwidth (full width at half maximum). All stimuli were generated with a program written in C++. We have set the center spatial frequency of the noise at 1.3 c/deg and the center orientation at 45 or -45 deg because we thought they were the easiest to look at the effects of spatial frequency bandwidth and of orientation bandwidth. For spatial frequency, the bandwidth was 1, 2, 3 or 4 octaves or unfiltered. For orientation, the bandwidth was 30, 60, or 90 deg or unfiltered. Here, unfiltered indicates that the amplitude spectrum was flat along that dimension. To make a comparison with preference or discomfort for 1/*f*^α^ noise reported in previous studies (Juricevic et al., 2010; Spehar and Taylor, 2013; Spehar et al., 2016, 2015; Viengkham and Spehar, 2018; Viengkham et al., 2019), we additionally employed 1/*f*^α^ noise with a slope (α) of 0.5, 1, 1.5, or 2. All stimuli were presented within a circular window with a 4.6 deg diameter. The edge of window was tapered by a cosine-wave with a wavelength of 1.1 deg. The RMS contrast of all stimuli was fixed to 0.3, and mean luminance was equated to that of the uniform background (111 cd/m^2^).

#### Procedure

We measured the degree of unpleasantness for each stimulus by using a rating scale method. On each trial, the stimulus was chosen randomly from the image set created in advance (48 images) and was presented for 500 ms. This duration was chosen because it appeared to minimize both strong impression of flashing and prolonged local adaptation. Observers freely viewed the stimulus and answered the question “Was the noise unpleasant?” on a 9-point scale that varied from “Not at all” (0) to “Very” (8). The stimulus in the next trial was presented 500 ms after the observer’s response. The average reaction time across all observers was about 950 ms. All instructions were given in Japanese. Almost none of the stimuli were rated as 0 by any observers. For each observer, 5 trial data were collected and averaged for each stimulus condition.

### Results

**FIGURE 2:**
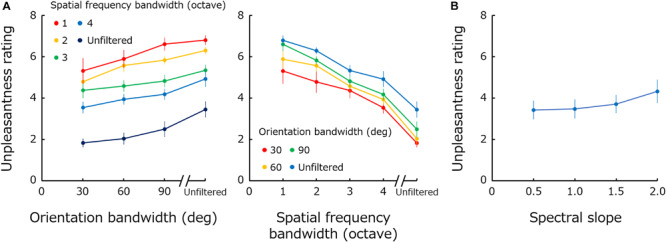
**(A)** Mean unpleasantness rating as a function of orientation bandwidth. Different colors represent results for different spatial frequency bandwidths. The right panel shows the same data replotted against spatial frequency bandwidth, and different colors represent the results for different orientation bandwidths. **(B)** Mean unpleasantness rating obtained for 1/*f*^α^ noise as a function of spectral slope (α). Error bars represent +-1 SE.

The left panel in Figure 2A plots mean unpleasantness rating across observers as a function of orientation bandwidth. Different colors represent results for different spatial frequency bandwidths. The right panel shows the same data replotted against spatial frequency bandwidth, and different colors represent the results for different orientation bandwidths. Figure 2B shows the rating data obtained for 1/*f*^α^ noise as a function of spectral slope (α).

A two-way repeated-measure ANOVA performed with spatial frequency bandwidth and orientation bandwidth as factors showed significant main effects of both frequency bandwidth [*F*(4,44) = 30.34; *p* < 0.0001; η_G_^2^ = 0.54] and of orientation bandwidth [*F*(3,33) = 6.86; *p* = 0.0010; η_G_^2^ = 0.16], and no significant interaction [*F*(12,132) = 1.19; *p* = 0.30; η_G_^2^ = 0.015]. A one-way ANOVA performed for the spectral slope showed a main effect of spectral slope [*F*(3,33) = 8.85; *p* = 0.0002; η_G_^2^ = 0.044].

### Discussion

These results show that unpleasantness is more profound if spatial frequency bandwidth is narrower and orientation bandwidth is broader. The results obtained for spatial frequency are qualitatively consistent with previous data showing that a positive bump in the spatial frequency spectrum (especially at 1–5 c/deg) gives rise to discomfort (Wilkins et al., 1984; Wilkins, 1995; Fernandez and Wilkins, 2008; O’Hare and Hibbard, 2011). While the perceived contrast of stimuli is different across spatial frequencies (Figure 1A), previous studies have shown that visual discomfort for middle spatial-frequency stimuli cannot be attributed to the difference in the perceived contrast (O’Hare and Hibbard, 2011). The visibility of high spatial frequency components may be rather a more important additional factor since the previous finding showed that spatial blur, or loss of high-spatial frequency information, in the image is associated with discomfort (O’Hare and Hibbard, 2013). The results obtained for orientation bandwidth appear to be opposite of those for spatial frequency. Stimuli with a flat orientation spectrum are perceived as the most unpleasant.

The results for 1/*f* noise show that the effect of the spectral slope on unpleasantness ratings is much smaller compared to the effect of spatial frequency or orientation bandwidths. This result demonstrates how remarkable the effect of modulations in the Fourier spectrum on visual unpleasantness is. Furthermore, the unpleasantness rating monotonically increases, though very weakly, with spectral slope – a result inconsistent with the previous finding that suggested an optimal slope of approximately 1 (Spehar et al., 2003, 2016, 2015; Juricevic et al., 2010; Spehar and Taylor, 2013; Viengkham and Spehar, 2018; Viengkham et al., 2019). These differences may simply due to the fact that these measurements are not comparable to the unpleasantness: preference, aesthetics or discomfort.

## Experiment 2

Experiment 1 showed that the unpleasantness increases as spatial frequency bandwidth becomes narrower and orientation bandwidth becomes broader. Since narrower bandwidth are most different from a flat spectrum, results also suggest that deviations from flat spectra are related to unpleasantness. If the deviation from a flat spectrum is critical, then similar patterns of the results should be found when the spectrum is modulated with multiple peaks (e.g., at 45 and 135 deg in case of orientation). To test this prediction, we generated noise textures whose amplitude spectra were sinusoidally modulated along orientation or spatial frequency by using a method from a previous study (Motoyoshi and Kingdom, 2003b).

### Methods

**FIGURE 3:**
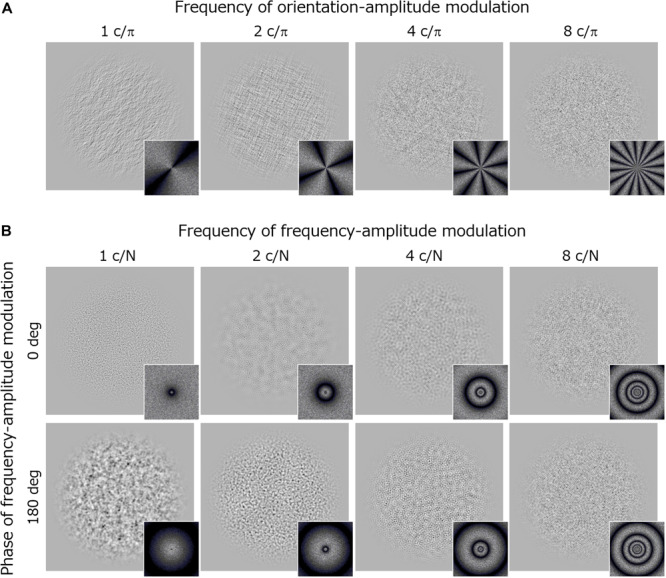
Stimuli and their Fourier spectra used in Experiment 2. **(A)** Orientation modulated noise textures with different modulation frequencies. **(B)** Spatial-frequency modulated noise textures with different modulation frequencies. Upper images show stimuli with a modulation phase of 0 deg and the lower images with a modulation phase of 180 deg.

Visual stimuli were noise textures (256 × 256 pixels) whose log-amplitude spectra were sinusoidally modulated from the 1/*f* baseline function along either the orientation or spatial frequency dimension (Figure 3).

The orientation modulated noise had an amplitude spectrum *A*(*f*, θ) that was sinusoidally modulated along the orientation dimension (θ) around the 1/*f* baseline. Thus, the noise was made by applying a filter following the equation:

$$\log\left(A\,\left(f,\theta \right)\right)=-\alpha \cdot f\cdot \left[1+M_{\text{ori}}\cdot \text{cos}\,\left(2\,\pi \,f_{\text{ori}}\cdot \,\frac{\theta }{\pi }\,+\varphi_{\text{ori}}\right)\right]$$

where *f* is the spatial frequency (c/image) in octave units, θ is the orientation in radian units, α is the slope of 1/*f* noise (fixed at 1.0), and *M*_ori_ is the amplitude of the orientation modulation. The stimulus is an unmodulated 1/*f* noise if *M*_ori_ is 0. *f*_ori_ is the frequency of the orientation-amplitude modulation (c/π), which took values of 1, 2, 4, and 8 in the experiment. φ_ori_ is a phase component that was decided randomly on each trial.

The frequency modulated noise had an amplitude spectrum *A*(*f*, θ) that was sinusoidally modulated along the log-spatial frequency (*f*) around the 1/*f* baseline. Thus, the noise was made by applying a filter following the equation:

$$\log\left(A\,\left(f,\theta \right)\right)\,=-\alpha \cdot f\cdot \left[1+M_{\text{freq}}\cdot \cos\left(2\,\pi \,f_{\text{freq}}\cdot \,\frac{f}{N}\,+\,\varphi_{\text{freq}}\right)\right]$$

where *f* is the spatial frequency (c/image) in octave units, and *N* is the sampling frequency (*N* = 8). α is the slope of 1/*f* noise (fixed at 1.0), and *M*_freq_ is the amplitude of the frequency modulation. *f*_freq_ is the frequency of the spatial-frequency modulation (c/N) and it took values of 1, 2, 4, and 8 in the experiment. φ_freq_ is a phase component that took values of either 0 or π (180 deg).

Eleven naïve paid volunteers and one of the authors (NO) took part in the experiment (6 females and 6 males, 5/12 participated in Expt. 1, aged 18–24). No one of them has a history of migraines. On each trial, the stimulus was chosen randomly from stimuli previously created (12 stimuli) and was presented for 500 ms. For each observer, 13 trial data were collected and averaged for each stimulus condition. The average reaction time in each trial was ∼1,100 ms on average across observers. Experimental conditions (other than those mentioned above) remained the same as in Experiment 1.

### Results

**FIGURE 4:**
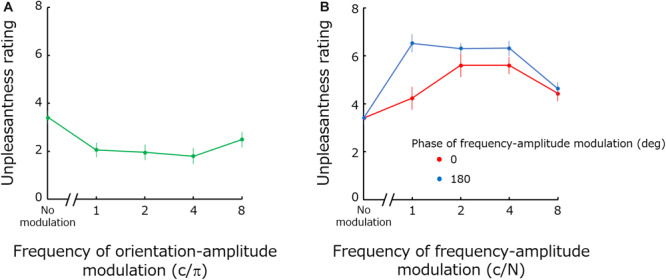
Mean unpleasantness ratings as functions of orientation modulation frequency **(A)** and the spatial-frequency modulation frequency **(B)**. Error bars represent +-1 SE.

Figure 4 shows mean unpleasantness rating as a function of orientation modulation frequency (a), and of spatial-frequency modulation frequency (b). Red and blue circles in Figure 4B show the results obtained for the frequency modulation phases of 0 and 180 deg -respectively.

The one-way repeated-measure ANOVA for the orientation modulated noise showed a significant main effect of modulation [*F*(4,44) = 6.18; *p* = 0.0005; η_G_^2^ = 0.23]. A two-way repeated-measure ANOVA for spatial-frequency modulated noise showed significant main effects both for the phase [*F*(1,11) = 7.95; *p* = 0.017; η_G_^2^ = 0.097] and for modulation frequency [*F*(4,44) = 17.70; *p* < 0.0001; η_G_^2^ = 0.40] as well as a significant interaction between the two factors [*F*(4,44) = 4.869; *p* = 0.0021; η_G_^2^ = 0.10].

### Discussion

As expected, modulating orientation reduces visual unpleasantness for a wide range of modulation frequencies (1–4 c/π) except at the highest frequency (8 c/π). A previous study has shown that detection sensitivity (1/threshold) for orientation modulation gradually increases up to 4 c/π and then rapidly decreases beyond that point (Motoyoshi and Kingdom, 2003b). Accordingly, it is likely that the lack of effect for high modulation frequencies (i.e., 8 c/π) is simply because the modulation at this frequency was not clearly perceived even at a suprathreshold level. Our results indicate that energy modulations – not only energy concentration (Expt. 1) – along the orientation dimension render the stimuli more comfortable.

Similarly, spatial frequency modulation increases unpleasantness for frequencies up to 4 c/N. According to the data from the conference abstract (Motoyoshi and Kingdom, 2003a) related with the study of orientation-energy modulation, detection sensitivity for spatial-frequency modulation also rapidly declines down after 4 c/N. Accordingly, high frequency modulations should be less perceivable. We also find a significant difference in the results between modulation phases along the spatial-frequency dimension. Modulations with a 180 deg phase have a larger impact than modulations with 0 deg phases, especially for lower modulation frequencies. We interpreted this as indication that 180 deg phase modulations (but not 0 deg phases) matched the optimal range of the (carrier) spatial frequency causing discomfort (1–6 c/deg) (Wilkins et al., 1984; Wilkins, 1995; Fernandez and Wilkins, 2008; O’Hare and Hibbard, 2011). Then 180 deg phase modulation gave rise to unpleasantness more profoundly than 0 deg phase modulation. Upon inspection of spectral distributions for the two phases, however, we find no clear evidence supporting this idea. Also, we found that perceived contrast can provide an explanation for the differing phase results. The perceived contrast of noise images depends on stimulus parameters. Especially, noises with 180 deg phase have higher perceived contrast compared to those with 0 deg phase. The unpleasantness rating may be just dependent on perceived contrast, but it should be noted that the relationship between perceived contrast and visual discomfort / unpleasantness is not so simple (O’Hare and Hibbard, 2011).

## General Discussion

The present study investigated visual unpleasantness by using noise textures with parametrically manipulated orientation and spatial frequency spectra. Experiment 1 showed that visual unpleasantness for bandpass noises decreases with spatial frequency bandwidth but increases with orientation bandwidth. In Experiment 2, we employed noise patterns whose Fourier amplitudes were modulated according to a sinusoidal waveform along either the spatial frequency or orientation dimensions. We found that, as long as modulations are clearly perceived, modulation along the spatial frequency dimension increases visual unpleasantness but modulation along the orientation dimension decreases visual unpleasantness.

Results from our two experiments indicate opposite directions for the effects of spatial frequency and orientation. Noise images with biased spatial-frequency spectra or flat orientation spectra tend to be judged as unpleasant. While these opposite trends appear to paint different pictures, we can give them a unified account by considering natural image statistics. As mentioned in the Introduction, the spatial-frequency amplitude spectrum of natural scenes decreases linearly in log-log coordinates. Conversely, however, orientation spectrum is rarely flat (Switkes et al., 1978; Van Der Schaaf and Van Hateren, 1996; Hansen and Essock, 2004). Thus, both results are consistent with the notion that humans feel unpleasant with images whose Fourier spectrum deviates from the statistical regularity of natural scenes. This is true for stimuli with a single (Expt. 1) and multiple (Expt. 2) spectral peaks.

The results from Expt. 1 obtained with spatial frequency are in agreement with previous data showing that images look relatively uncomfortable if their amplitude spectra have a peak at spatial frequencies of 1.5–6 c/deg or 0.4–1.5 c/deg (Fernandez and Wilkins, 2008; O’Hare and Hibbard, 2011). A similar range of spatial frequencies (1–4 c/deg) has been linked to paroxysmal excitation (Wilkins et al., 1980; Shepherd, 2001). The center spatial frequency of our bandpass noise in Expt. 1 (1.3 c/deg) is certainly within this range. Studies with natural images have shown that middle spatial frequencies relative to the image size, e.g., 4–16 c/image, are correlated with trypophobic responses (Cole and Wilkins, 2013; Le et al., 2015). This spatial-frequency range is also consistent with our noise stimuli (8 c/image), but it is unclear which spatial frequency – retinal or relative – is critical. However, the results of Expt. 2 for spatial-frequency modulation are difficult to interpret. The data shown in Figure 4 indicate that the effect of modulation depends on phase and suggest that 180-deg phase modulation increased amplitudes at around the above frequency range (∼3 c/deg), but we did not find any evidence for it.

Although the physiological basis of effects we have found is unclear, our results may be interpreted in relation with the neural mechanisms of a visual brain that adapts to its natural environment (Simoncelli and Olshausen, 2001; Clifford et al., 2007). Because the human visual system is most responsive to spatial frequencies at around 3 c/deg (Campbell and Robson, 1968), it has been suggested that visual discomfort from images modulated along frequency is associated with excessive energy consumption triggered by stimuli in that spatial frequency range (Fernandez and Wilkins, 2008). This theory can also explain reduced discomfort/unpleasantness in images with broad spatial frequency spectra (e.g., 1/*f*), in which the response of simple and complex cells in V1 are mutually suppressed across spatial frequencies due to contrast normalization processes (Heeger, 1992; Schwartz and Simoncelli, 2001). However, this account is inconsistent with unpleasantness in images with flat orientation spectra because the responses of these cells are mutually suppressed across orientations too. An alternative metabolic account that may reconcile the discrepancy is that unpleasantness involves neurons with non-oriented receptive fields that are most responsive to images with flat orientation spectra but less to images to flat spatial frequency spectra. Indeed, such neurons are concentrated in CO blobs in V1 and exhibit excessive energy (oxygen) consumption (Livingstone and David, 1984, 1988; Bartfeld and Grinvald, 1992).

Aside from the metabolic explanation, another hypothesis suggests that the stimulus specificity of unpleasantness simply reflects the response property of sub-cortical pathways – including superior colliculus and pulvinar – involved in the rapid and direct emotional response to threatening stimuli such as snakes (Van Le et al., 2013; Ahmadlou and Heimel, 2015; Méndez-Bértolo et al., 2016; McFadyen et al., 2017). The majority of visual neurons in this pathway are known to have isotropic receptive fields tuned to a middle spatial frequency band of approximately 1 c/deg (Chen et al., 2018). A study also suggested the involvement of V2 in painful discomfort by grating especially among migraine patients (Huang et al., 2011), but it is unclear how this finding is related with the unpleasantness as examined in the present study.

The present results, together with the previous data (Chatrian et al., 1970; Wilkins, 1995; Conlon et al., 2001; O’Hare and Hibbard, 2011; Penacchio and Wilkins, 2015; Sasaki et al., 2017), indicate a clear effect of an image’s Fourier spectrum on visual discomfort/unpleasantness. Caution is warranted, however, because we cannot rule out that the reported effects are caused by other stimulus properties, e.g., higher-order features, that can be correlated with spectral properties. Nevertheless, we believe that it would be useful to investigate the effects of such low-level image statistics. Indeed, recent studies are investigating the various emotional responses for visual stimuli for a variety of image statistics including skewness, kurtosis, correlations across subbands, temporal frequency (Graham et al., 2016; Yoshimoto et al., 2017).

## Data Availability Statement

The datasets generated and analyzed during the current study are available from the corresponding author upon reasonable request.

## Ethics Statement

The studies involving human participants were reviewed and approved by the Ethics Committee for Experiments on Humans at Graduate School of Arts and Sciences, The University of Tokyo. The patients/participants provided their written informed consent to participate in this study.

## Author Contributions

IM conceived the study. NO and IM designed the experiments and wrote the manuscript. NO collected and analyzed the data.

## Conflict of Interest

The authors declare that the research was conducted in the absence of any commercial or financial relationships that could be construed as a potential conflict of interest.
